# A Molecular Dynamics Study of the Mechanical Properties of Twisted Bilayer Graphene

**DOI:** 10.3390/mi9090440

**Published:** 2018-08-31

**Authors:** Aaron Liu, Qing Peng

**Affiliations:** 1 Computer Sciences and Engineering, University of Michigan, Ann Arbor, MI 48109, USA; aaronliu@umich.edu; 2 Nuclear Engineering and Radiological Sciences, University of Michigan, Ann Arbor, MI 48109, USA; 3 Department of Mechanical, Aerospace and Nuclear Engineering, Rensselaer Polytechnic Institute, Troy, NY 12180, USA

**Keywords:** bilayer graphene, mechanical properties, stress-strain, fracture toughness, strain rate

## Abstract

Graphene is one of the most important nanomaterials. The twisted bilayer graphene shows superior electronic properties compared to graphene. Here, we demonstrate via molecular dynamics simulations that twisted bilayer graphene possesses outstanding mechanical properties. We find that the mechanical strain rate and the presence of cracks have negligible effects on the linear elastic properties, but not the nonlinear mechanical properties, including fracture toughness. The “two-peak” pattern in the stress-strain curves of the bilayer composites of defective and pristine graphene indicates a sequential failure of the two layers. Our study provides a safe-guide for the design and applications of multilayer grapheme-based nanoelectronic devices.

## 1. Introduction

Graphene, a single layer of carbon atoms arranged in a hexagonal lattice, is known for its extraordinary physical, chemical, and mechanical properties [1,2,3,4,5,6,7,8,9]. There have been extensive investigations for the fundamental properties and potential applications of graphene since its recent discovery and synthesis in experiments [10,11]. For example, the small spin-orbit interaction of graphene makes it an ideal material for spintronics [12]. The extraordinary sensitivity makes it a great material for chemical sensors, since its electronic properties are highly susceptible to the absorption of gaseous atoms [13]. The extraordinary electronic properties, such as high electron mobility under room temperature [14], make graphene ideally suited for the fabrication next generation logic device [15]. Graphene’s ultra-high intrinsic strength [16] make it a good candidate as reinforcement [17]. For instance, a graphene/poly nano-composite material is reported to give a 76% increase in tensile strength and a 60% increase in Young’s modulus [18]. Graphene is also a promising additive to enhance the radiation damage resistance for structural nuclear materials [19]. Its superior mechanical properties make graphene a great material for nano-electro-mechanical system (NEMS) applications. The robust, strong, and stable structure of single-layer graphene allows it to be made into nano-resonators after being suspended [20]. In addition, graphene has promising applications in flexible transparent conductors in smart windows, phones, etc. [21,22], as well as in the optical domain, such as graphene photonics and plasmonics [23,24,25,26].

Although graphene has such superior properties and great applications, it has a zero band gap, which prevents it from being a suitable alternative to silicon in the electronics industry [27]. It has been reported that the electronic bandgap can be tuned in bilayer graphene [28]. A twisted graphene bilayer is a group of two graphene monolayers stacked together, with a mutual disorientation with a finite angle ((0° < γ < 30°), rather than AA stacked (γ = 0°) or Bernal stacked (γ = 30°). The second layer is parallel to the first layer, but shifted, which is the same layered structure as the graphite, but only two layers. The unique electronic properties triggered great research interest in the bilayer graphene moire superlattice [29,30,31]. A recent experimental investigation reported that electron transfer rate and chemical reaction rate can be significantly modified in twisted bilayer graphene with the twist angle of 25.2° [32]. Recent theories have shown that the electronic band structures of the two graphene layers can be modified by the interlayer coupling between them [33,34,35], which allows graphene to be a silicon alternative in the electronics industry. In addition, it provides a way for the modification of electron transfer rates.

There is a decent amount of studies on twisted graphene bilayers [29,30,31,32]. However, most of them focus on the differences in the electronic properties and chemical properties. The mechanical properties of twisted bilayer graphene were previously studied using first-principles calculations at zero temperature [36]. To the best of the authors’ knowledge, the fracture toughness of twisted bilayer graphene remains unexplored until this work, which is important for its applications in the electronics industry [37,38,39]. Here, a comprehensive study of twisted bilayer graphene is presented using molecular dynamics (MD) simulations. The mechanical properties of twisted bilayer graphene are investigated, including the stress-strain relationships and the fracture toughness. We focus this study on the one case of twist angle of 25.2° because this one has been experimentally investigated with five times faster electron transfers and chemical reactions [32], but the mechanical properties are not still not clear.

## 2. Models and Methods

The MD simulations were carried out using the Large-scale Atomic/Molecular Massively Parallel Simulator (LAMMPS) [40] software package (Sandia National Laboratories, Livermore, CA, USA), which features a classical molecular dynamics code. The force-field that describes the interactions between carbon atoms is the adaptive intermolecular reactive bond order (AIREBO) [41], which allows for covalent bond breaking and forming, enabling an accurate description of interactions between atoms under extreme conditions, including fracture. It is well known that the potential of the original AIREBO is not good for the fracture of graphene; we used the modified version [42] in our study, which has also been used to study the mechanical phase change of graphene with verifications [43]. The initial configuration of a single layer graphene sheet was built, with a C–C bond length of 0.142 nm. The first layer of graphene exhibited armchair edges along the *x*-axis, and zigzag edges along the *y*-axis. The second layer of graphene was shifted parallel relative to the first layer, with a disorientation angle γ = 25.2°. The primitive unit cell of such a twisted-bilayer contained 532 carbon atoms. The model was periodic along the two in-plane directions (*x* and *y*), and the third direction was fixed (*z*). We used a 4 × 4 × 1 super cell (8512 atoms) in this study to reduce the self-image interactions of the defects.

**Figure 1:**
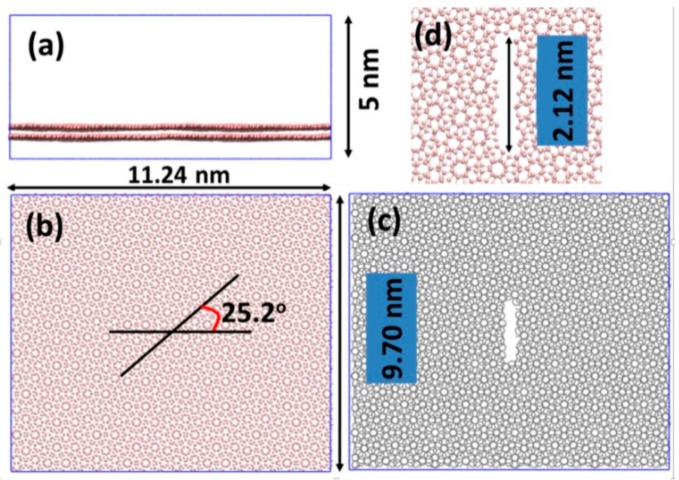
Simulation box and configurations of twisted bilayer graphene. (**a**) Side and (**b**) top views of the simulation box, respectively. The *x*, *y* axes are the horizontal and vertical axes, respectively. (**c**) Systems with pre-crack. (**d**) The zoom-in plot of the pre-crack. The two layers of graphene are twisted, with an angle of 25.2°.

All the simulations were carried out at a temperature of T = 300 K. All systems were fully equilibrated at a pressure of atmosphere (P = 0.0001 GPa) using the isothermal–isobaric ensemble (constant temperature and constant pressure ensemble, or NPT ensemble) before any mechanical loading. The simulation time step was 0.0005 picoseconds (ps). The size of the equilibrated simulation box was 11.24 × 9.7 × 5 nm^3^, and contained 8512 carbon atoms as shown in Figure 1. To characterize the mechanical properties, tensile loading was applied along the x direction that was perpendicular to the pre-cracks, as shown in Figure 1c. The pre-cracks were obtained by removing atoms in specific areas. The pre-cracks in this study were perpendicular to the *x* axis (the horizontal axis), which is denoted as an armchair direction, in line with the literature. All the strain, stress, and the Young’s modulus, were specified to this direction in this study.

From the simulated stress–strain curves, the Young’s modulus E, strength σ, and fracture strain $\varepsilon_{F}$ could be obtained; the Young’s modulus was calculated as the initial slope of the stress–strain curve; the strength and fracture strain were defined at the point where the peak stress was reached. All the strain in this study were engineering strain. 

## 3. Results and Discussion

### 3.1. Structural Properties and Bulk Moduli

We firstly relaxed the twisted bilayer graphene using the isothermal–isobaric ensemble (NPT) for 550 ps with T = 300 K and P = 0.0001 GPa (representing ambient condition), to remove the residual stress of the system. The equilibrated system is depicted in Figure 1. The side-view of the system shows that the bi-layer graphene is not flat, but instead contains wiggles (Figure 1a). The average projected area per carbon atom in the simulation box was 2.5588 × 10^−2^ nm^2^, which corresponded to the C–C bond length with a component of 0.1405 nm parallel to the projected plane (*x*-*y* plane). It was slightly smaller than the experiment value of 0.142 nm and the original AIREBO value of 0.141 nm. Such a 0.46% contraction, referring to the ideal theoretical potential, indicates a tilt angle of 0.26°, due to out-of-plane buckling.

**Figure 2:**
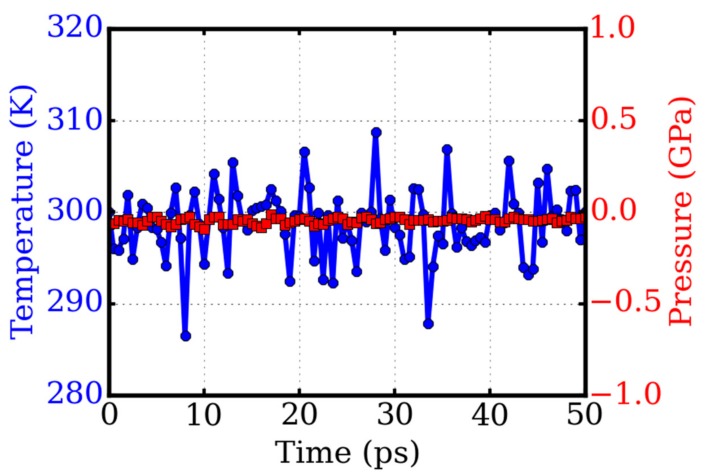
System equilibration with isothermal–isobaric (NPT) ensembles. The target temperature and pressure is 300 K and 0.0001 GPa, respectively. The bulk modulus of the twisted bilayer graphene is 1.48 TPa calculated from the thermal fluctuations in equilibration.

The temperature and pressure of the system fluctuated around the target value after equilibrium. The thermal fluctuations of the last 50 ps of the equilibration are shown in Figure 2. The average value was 298.7 K and −0.047 GPa, with a standard deviation of 3.6 K and 0.02 GPa for temperature and pressure, respectively. The average volume was $V_{0}$ = 134.8 nm^3^, with a standard deviation of $\sigma_{V}$ = 0.016 nm^3^, while the thickness of 5 nm was fixed. The bulk modulus $B_{0}$ could then be obtained from the fluctuations of the pressure and volume through the formula $B_{0}=k_{B}\;T\frac{V_{0}}{\sigma_{V}^{2}}$, where $k_{B}$ is the Boltzmann constant. We had $B_{0}$ = 1.48 TPa for this twisted bilayer graphene, which compares well with the stiffness of monolayer graphene, as 1.2 TPa [20].

It is worth mentioning that the graphene monolayer was non-isotropic. The twisted-bilayer graphene was in principle also non-isotropic and influenced by the twist angle. However, this angle-dependence should be less than that of monolayer graphene. The angle-dependent properties in bilayer graphene deserve further studies. 

### 3.2. The Stress–Strain Relationship

**Figure 3:**
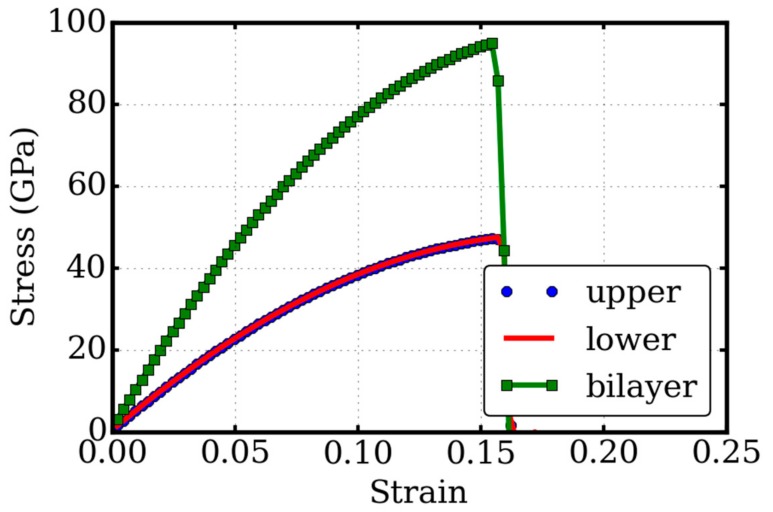
Tensile tests of twisted-bilayer graphene. The stress–strain relationship of the twisted bilayer graphene (bilayer) is compared with single layer graphene of upper part (upper) and lower part (lower). All the thicknesses are set to 0.668 nm for the convenience of comparison.

The stress–strain relationship plays an essential role in the characterization of mechanical properties of a material or structure. The stress-strain relationship is, in general, obtained from tensile tests. To model the tensile tests, we elongated the simulation box in the direction. The strain was measured as “true” strain, which means that the box dimension changes non-linearly with time from its initial to final value. The variation of the simulation box length as a function of time was described as *L*(*t*) = *L*_0_ exp(*η* × *t*), where *L*(*t*) is the length of the simulation box at time *t*, *L*_0_ is the initial length, and *η* is the true strain rate, in unit of s^−1^. Thickness is one of the most challenge questions for dealing with the mechanical properties of atomic-thick 2D materials, because their thickness (third-dimension) is not well defined, which is totally different from conventional bulk materials. For the convenience of comparison, we set all the thicknesses to be 0.668 nm, which corresponded to the thickness of two atomic layers of carbons in bulk graphite. The tensile stresses were computed from the normal Cauchy stresses, multiplied by a factor of 7.485 which was the ratio of the thickness of the simulation box (5 nm) to the thickness of double layer graphene (0.668 nm). Our results of the tensile test modeling of the pure twisted bilayer graphene are illustrated in Figure 3, compared with those of single layer graphene in the upper layer and the lower layer.

In a small regime of strain of about a few percent, when the tensile strain increases, the stress that the system experienced enlarges linearly. Such linearity is measured by Young’s moduli, which is calculated as the initial slope of the stress–strain curve. Our stress–strain relationship revealed a Young’s modulus of 0.96 TPa. When a large strain was applied, the stress of the system responded non-linearly to the strain until the system’s failure. The ultimate tensile strength was the maxima in the stress-strain curve, indicating the upper strength limit of the system. The corresponding strain was defined as the ultimate tensile strain, which reflected the flexibility of the system. The ultimate tensile strength and strain were 94.9 GPa and 0.1545 respectively. 

Our results of the Young’s modulus, the ultimate tensile stress, and the strain indicated that the modulus of the twisted bilayer graphene was the same as the single layer graphene. To verify this point, we examined the stress–strain relationships of single layer graphene. The upper layer graphene behaved the same as the lower layer in the tensile tests, as illustrated in Figure 3. This study confirms that with the bonus of advanced electronic properties, there is no penalty to its outstanding mechanical characters. This indicates a greater range of applications for twisted bilayer graphene than monolayer graphene.

### 3.3. Fracture Toughness

Fracture toughness describes the ability of a material containing a crack to resist fracture. As an intrinsic property, fracture toughness is one of the most important mechanical properties of any material. Through tensile tests of the pure twisted bilayer graphene, we obtained the ultimate tensile stress which intrinsically governed the uniform breaking of atomic bonds (C–C bonds) in a perfect system. Considering the wide applications, the useful strength with engineering relevance is usually determined by its fracture toughness [44,45].

**Figure 4:**
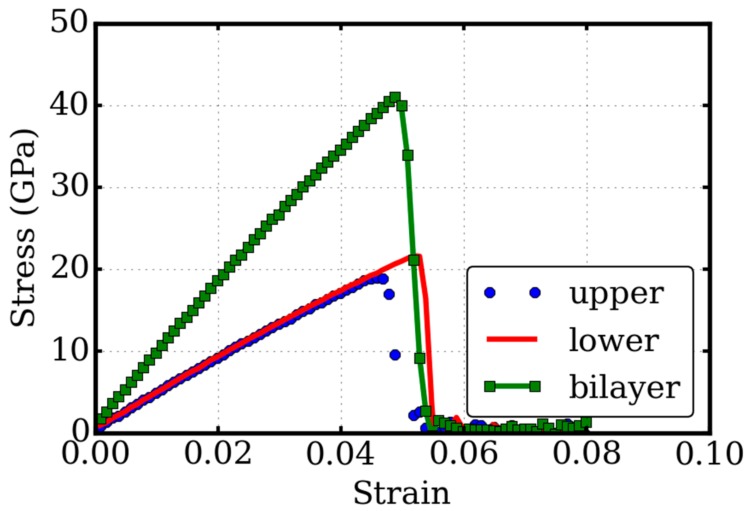
Tensile tests of the twisted bilayer graphene with the pre-crack. The stress–strain relationship of the twisted bilayer graphene (bilayer) is compared with the single layer graphene of the upper part (upper) and lower part (lower). All the thickness is set as 0.668 nm for the convenience of comparison. The pre-crack has the same shape and size in the three cases. The critical stress intensity factor of fracture is $K_{c}=2.4\;$ MPa$\sqrt{m}$.

We generated a pre-crack by removing atoms within a regime defined by a rectangular box of 0.305 × 2.0 × 5.0 nm^3^. A total of 48 carbon atoms were removed within this regime, 24 atoms on each layer. The length of the pre-crack measured by the remaining atoms was 2.12 nm, as shown in Figure 1c,d. After relaxation of the pre-cracked twisted bilayer graphene system, we performed the tensile testing simulations. The stress-strain relationships of the pre-cracked systems are illustrated in Figure 4. The pre-crack had the same shape and size in the three cases.

We observed that the Young’s modulus of the pre-cracked system was the same as the pure system, which indicates that the pre-crack had little effect on the linear elastic properties. However, the non-linear mechanical properties were very different. With the crack, the ultimate tensile stress reduced to 41.03 GPa, a drop of 57% from the perfect system, which was 94.9 GPa. The ultimate tensile strain also had a large (68.4%) drop, from 0.1545 to 0.0488. Such a large drop in ultimate stress is very much expected when a crack is present due to concentration of stresses (increase in compliance). In addition, the drop in peak stress seemed to be more or less similar in both the monolayer and twisted layer, which indicates that the physics was not all that different. This could be understood as the coupling between the two layers not being strong because of the bond nature of weak Van der Waals interactions between layers. 

Both the perfect and pre-cracked twisted bilayer graphene undergoes brittle failure at large mechanical loads. Once the crack is initiated, it will propagate across the whole system immediately and cause global failure. Our observation agrees well with the experimental investigation [44,45]. According to the classic Griffith’s theory, brittle fracture occurs when the strain energy exceeds the surface energy of the created surface for an infinitesimal extension of the crack. The Griffith criterion can be then expressed by the critical stress of the onset of fast fracture, as $\sigma_{c}=\sqrt{\frac{2\gamma_{s}E}{\pi a_{0}}}$, where $\gamma_{s}$ is the surface energy density, E is the Young’s modules, and $\;a_{0}=\frac{L}{2}$ is the half length of the crack slit. The fracture toughness is conventionally characterized by the critical stress intensity factor of the fracture as $K_{c}=\sigma_{c}\sqrt{\pi a_{0}}$. With $\sigma_{c}=41.03\;$ GPa and $\;a_{0}=1.06\;$ nm, we have $K_{c}=2.4\;$MPa$\sqrt{m}$, which roughly agrees with the experimental measurement of 4.0 MPa$\sqrt{m}$ [44]. The difference might be attributed to the small simulation box, and the high strain rate is limited in molecular dynamics simulations. Our fracture toughness is 9.54 J/m^2^, agreeing with MD studies in the literature, as reviewed in [20].

We have also examined the mechanical behaviors of the individual components of the two layers. The critical stress and strain are 18.98 GPa, 0.0458 and 21.58 GPa, 0.0528 for the upper and lower layer graphene, respectively. The differences might stem from the different orientations to the mechanical loading due to the anisotropic mechanical behaviors of graphene. However, such differences are “averaged” in the system, which indicates that there are still some strong couplings between layers. This reasoning is verified by the observation of inter-layer bonding around the critical loading.

### 3.4. System Size Effect

**Figure 5:**
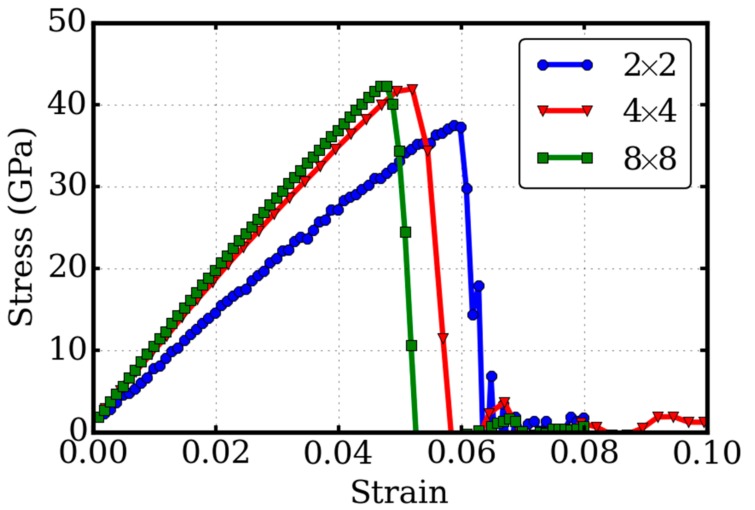
System size effect. Stress-strain relationships of the twisted bilayer graphene are tested in three super cells which contain 2 × 2, 4 × 4, and 8 × 8 unitcells.

When periodic boundary conditions are applied to a defective system, it is inevitable that the defects interact with its own periodic images or self-images, due to the long range of the elastic field. As a result, the system size in general has an effect on the defective system. To reduce this artificial effect, the system size should be large enough. The system size effect is examined in this study through three super cells which contain 2 × 2, 4 × 4, and 8 × 8 unitcells respectively. The number of carbon atoms are 2128, 8512, and 34,048, respectively before the generation of the pre-crack. Since the pre-cracks generated in all the three systems have the same siz e, the system size effect on the fracture toughness is equivalent to the effect on the critical stress $\sigma_{c}$. The simulations of the tensile tests on the three systems are carried out under the same conditions, as aforementioned. The results of the stress-strain relationships are illustrated in Figure 5.

As seen from Figure 5, the system size had an effect on the linear elastic properties if the system is small, indicating very strong interactions when the self-images were too close. The critical stresses were 37.51, 41.92, and 42.32 GPa for the small, medium, and large super cells, respectively. Our tests show that the results of the critical stresses converged to 42 GPa for sufficiently large systems. In other words, the system size effect on the fracture toughness is negligible for system sizes that are larger than the medium one. This test validated our results in the previous sections. 

### 3.5. Strain Rate Effect

**Figure 6:**
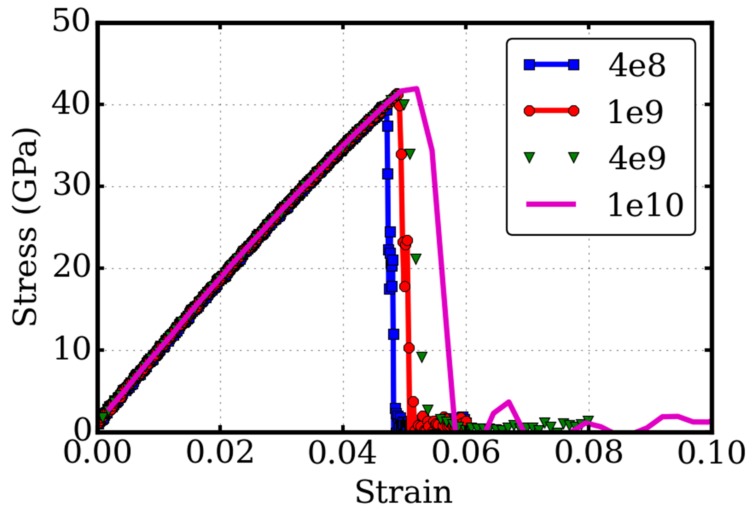
Strain-rate effect on the fracture toughness. Stress-strain relationship, as well as the fracture toughness of the twisted bilayer graphene with pre-crack, is examined in four strain-rates.

It is well known that the strain rate influences the mechanical properties, especially the fracture toughness, because it takes time for the system to respond to the applied mechanical loading. Due to computational resource limitations, the strain rates in the molecular dynamics simulations are in the order of 10^9^ s^−1^, much larger than those used in experimental studies, which are around 10^−3^–10^3^ s^−1^. The medium system of the pre-cracked twisted bilayer graphene was examined with tensile tests under four different strain rates: 4.0 × 10^8^, 1.0 × 10^9^, 4.0× 10^9^, 1.0 × 10^10^. The results of the stress-strain relationships are illustrated in Figure 6.

Our results show that the strain rate had little effect on the linear elastic properties, as depicted by the Young’s modulus. The critical stresses were 39.8, 41.3, 41.0, and 41.9 GPa, for the four strain rates, respectively. Although the strain rate differed by 25 times, there was no clear trend for the strain rate effect. In fact, at these high strain rates, the material had little time to respond to mechanical stimuli (adiabatic conditions). This is the reason for why almost all the results looked identical. Nevertheless, this study implied that the strain rate has negligible effect on the fracture toughness of the twisted bilayer graphene in molecular dynamics simulations. Further studies with low strain rate are interesting, but they are outside the scope of molecular dynamics simulations.

### 3.6. Layered Structure Effect

**Figure 7:**
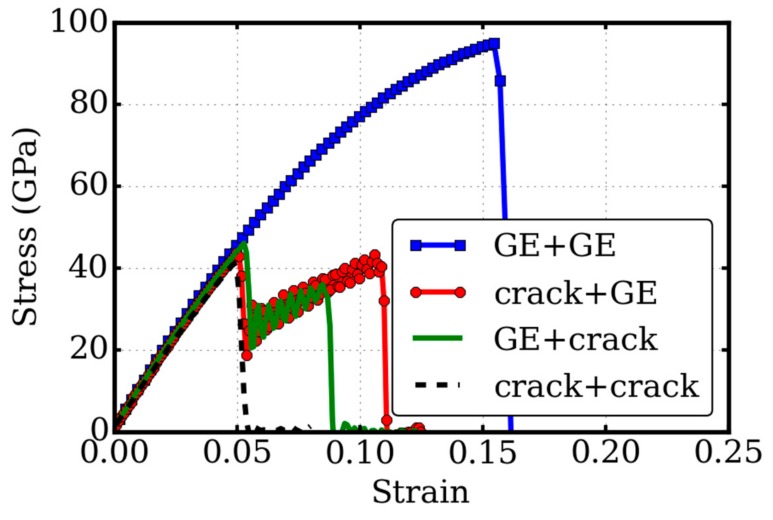
Layered structure effect. The stress–strain relationship of the twisted bilayer graphene is examined in four structures: pure twisted bilayer graphene (GE + GE), one pre-crack in upper layer (crack + GE), one pre-crack in lower layer (GE + crack), and pre-cracks in both layers (crack + crack). The hybrid structures have a two-peak pattern in the stress–strain curves, reflecting the sequential failure of the two layers.

Different from the monolayer graphene, the twisted bilayer graphene has structures with interlayer interactions. To examine this layered structure effects on the fracture toughness, we studied two additional hybrid structures or composites: the pre-crack that exists in only the upper or lower layers, denoted as “crack + graphene (GE)” and “GE + crack” respectively. The results of the stress-strain relationships from the tensile test simulations are illustrated in Figure 7, compared with those of the perfect twisted bilayer graphene (GE + GE) and the pre-cracked twisted bilayer graphene (crack + crack).

Our results demonstrated that all these cracks had little effect on the linear elastic properties, but a large effect on the fracture toughness and the flexibility. It is very interesting that there were two peaks in the stress–strain curves of the hybrid structures. For the “crack + GE” system, the two peaks were 43.7 and 43.4 GPa at the strains of 0.0498 and 0.1058. For the “GE + crack” system, the two peaks were 46.1 and 36.0 GPa at the strain of 0.0528 and 0.0848. Such distinctive characteristics suggest that there are different and complex fracture mechanisms in the hybrid structures. This difference might be attributed to the different orientations between the upper layer and the lower layer, referring to the tensile strain direction. The non-isotropic mechanical properties of graphene also affected the mechanical response of the composite with the heterogeneous structures. Nevertheless, the two hybrid structures shared the same patterns of double peaks in the stress–strain relationship, which clearly reflects the double-layered structure. The layer with a pre-crack failed first at a strain of about 0.05. The other graphene layer without a pre-crack still maintained system integrity, and the loading could be further sustained, until the failure of the second layer. The two-peak pattern revealed the sequential failure of the two layers. Our results of the two-peak pattern in the stress-strain curves suggest a new method to detect the number of layers in the multilayered structures via tensile tests.

It is worth noting that the twisted bilayer graphene in this study was in a “free-standing” state without interaction with other objects. However, in most of the applications involving graphene, a substrate is in inevitable, which will affect these twisted bilayer graphene mechanics. The extent to which this occurs depends on the coupling between the substrate and the graphene. In general, such an interaction is through Van der Waals interaction, and it is small. A thorough study of the substrate influence is desirable for real applications of this twisted bilayer graphene.

## 4. Conclusions

We have investigated the mechanical properties of twisted bilayer graphene via molecular dynamics simulations. The bulk modulus of twisted bilayer graphene is similar to the single layered graphene. The bilayer graphene has double the stiffness of single layered graphene, as implied in the stress–strain relationship. The system size effect on the fracture toughness is examined. The strain rate has negligible effect on the fracture toughness. The linear elastic properties are insensitive to the defects and strain rates, which are opposite to the non-linear mechanical properties, including fracture toughness. The hybrid structures combined with the defective (crack) and pristine graphene layers demonstrated a “two-peak” pattern in the stress-strain curves, indicating a sequential failure of the two layers, suggesting a method for characterizing the layered structure via tensile tests. Compared to the single layered graphene, the twisted graphene bilayer retains its extraordinary mechanical properties with the extra bonus in advanced electronic and optical properties, showing a great promise in a wide range of applications.
